# Determinants of Speech Perception Outcomes After Hearing Aid Fitting in Conductive and Sensorineural Hearing Loss: A Prospective Longitudinal Observational Study

**DOI:** 10.3390/audiolres16030086

**Published:** 2026-06-03

**Authors:** Akmaral Izbassarova, Assel Imangaliyeva, Vigen Bakhshinyan, Rimma Suatbayeva, Zilola Mavlyanova, Assel Izbassarova, Murat Auyelbayev, Kanat Kumar, Aizhan Aidaralieva

**Affiliations:** 1 Department of Physical Medicine and Rehabilitation Sports Medicine, Asfendiyarov Kazakh National Medical University, Almaty 050012, Kazakhstan; izbassarova.a@kaznmu.kz; 2 Department of Otorhinolaryngology, Almaty Multidisciplinary Clinical Hospital, Almaty 050009, Kazakhstan; alenarsentai2009@gmail.com; 3 Department of Clinical Audiology, Russian Medical Academy of Continuous Professional Education, 125040 Moscow, Russia; bakhshinyan@yahoo.com; 4 Saint-Petersburg Research Institute of Ear, Throat, Nose and Speech, 190013 Saint-Petersburg, Russia; 5 Department of Otorhinolaryngology, Asfendiyarov Kazakh National Medical University, Almaty 050012, Kazakhstan; 6 Department of Medical Rehabilitation, Sports Medicine and Traditional Medicine, Samarkand State Medical University, Samarkand 100400, Uzbekistan; reab.sammi@mail.ru; 7 Multidisciplinary City Children’s Hospital N3, Astana 010000, Kazakhstan; asko1199@mail.ru; 8 City Clinical Hospital N5, Almaty 050051, Kazakhstan; info@gkb5.kz; 9 Department of Otorhinolaryngology, Medical Center “Estu Sezim”, Aktobe 030007, Kazakhstan; kumarkanat1981@gmail.com; 10 Department of Science, Asfendiyarov Kazakh National Medical University, Almaty 050012, Kazakhstan; aidaralieva.a@kaznmu.kz

**Keywords:** hearing loss, sensorineural hearing loss, conductive hearing loss, hearing aids, speech perception, speech-in-noise, auditory rehabilitation, audiology, longitudinal study, Kazakhstan

## Abstract

**Background/Objectives:** Hearing loss is a leading cause of disability worldwide, with speech perception representing a key functional outcome of auditory rehabilitation. While hearing aids improve audibility, outcomes vary substantially across clinical subgroups. This study aimed to compare speech perception outcomes after hearing aid fitting in adults with conductive and sensorineural hearing loss and to identify determinants of variability in rehabilitation outcomes. **Methods:** This prospective longitudinal observational study included 250 adults with clinically confirmed bilateral conductive or sensorineural hearing loss who underwent standardized audiological assessment, bilateral hearing-aid fitting, immediate post-fitting evaluation, and 3-month follow-up in Kazakhstan between January 2023 and December 2024. Participants were classified as having conductive (*n* = 100) or sensorineural hearing loss (*n* = 150) based on audiometric criteria. Speech perception was assessed using a Kazakh-language open-set speech audiometry test. Multivariable linear regression models were used to estimate differences in 3-month aided speech perception after adjustment for the corresponding immediate post-fitting aided score and prespecified demographic, clinical, and audiometric covariates. Linear mixed-effects models were used separately to assess change in aided speech perception from immediate post-fitting to 3 months and to test whether this change differed by hearing-loss type. Propensity score matching was performed as a secondary sensitivity analysis. **Results:** Patients with conductive hearing loss demonstrated consistently higher speech perception scores than those with sensorineural hearing loss across all conditions. At 3 months, adjusted analyses showed no significant difference between groups for aided speech perception in quiet at 60 dB SPL, whereas sensorineural hearing loss remained associated with lower aided speech perception in noise at 60 dB SPL with SNR +3 dB (β = −1.73; 95% CI: −3.10 to −0.36; *p* = 0.014). In mixed-effects models assessing repeated aided scores from immediate post-fitting to 3 months, sensorineural hearing loss was associated with lower overall aided speech perception in both quiet and noise conditions. A modest improvement over time was observed only for speech perception in noise, and the group-by-time interaction was not statistically significant. Increasing age, higher tonal thresholds, advanced hearing loss stage, and living alone were independently associated with poorer outcomes. **Conclusions:** Aided speech perception scores were high after hearing-aid fitting in both conductive and sensorineural hearing loss; however, patients with sensorineural hearing loss showed persistently poorer outcomes, particularly in noise. These findings highlight the importance of incorporating speech-in-noise assessment and addressing clinical and social determinants to support hearing rehabilitation.

## 1. Introduction

Hearing loss is a major public health challenge, particularly in aging populations. The Global Burden of Disease 2021 study estimates that more than 1.5 billion people worldwide experience some degree of hearing loss, a figure projected to rise substantially as the global population continues to age [1,2]. Hearing loss is especially prevalent in countries with a middle sociodemographic index. By 2050, more than 2.1 billion individuals will be aged 60 years or older—nearly double the number recorded in 2020, according to the World Health Organization [3].

Among the different types of hearing impairment, conductive and sensorineural hearing loss differ fundamentally in their underlying pathophysiology, clinical presentation, and response to rehabilitation strategies [4,5,6]. While conductive hearing loss is typically related to middle ear dysfunction and often amenable to medical or surgical correction [7], sensorineural hearing loss involves damage to the cochlea or auditory pathways and generally requires long-term rehabilitative interventions, most commonly through hearing aids [8,9].

Hearing aids remain the cornerstone of intervention in auditory rehabilitation [10,11]. However, their effectiveness varies substantially across individuals and clinical subgroups [12,13,14]. Speech perception in quiet and in noise is considered one of the most clinically meaningful outcomes in hearing aid users, reflecting real-world communicative function [15,16]. Previous studies have demonstrated that patients with conductive hearing loss often achieve higher speech perception scores compared with those with sensorineural hearing loss [17,18]. However, the extent to which these differences persist after hearing aid fitting and during early adaptation remains insufficiently characterized. There is also a lack of data from Central Asian populations, where healthcare systems, access to audiological services, and sociodemographic factors may differ from those in high-income countries, potentially influencing rehabilitation outcomes. These gaps limit the generalizability of existing evidence and hinder the development of context-specific clinical and public health strategies.

In this context, the present study was undertaken to provide a comprehensive evaluation of hearing aid–related speech perception outcomes in adults with conductive and sensorineural hearing loss in Kazakhstan. The primary aim of this study was to compare aided speech perception in quiet at 60 dB SPL and aided speech perception in noise at 60 dB SPL with SNR +3 dB at 3 months between patients with conductive and sensorineural hearing loss. Secondary aims included assessing baseline differences in unaided and aided speech perception, evaluating longitudinal changes over time, and identifying factors associated with variability in hearing aid outcomes.

## 2. Materials and Methods

### 2.1. Study Design and Study Population

This prospective longitudinal observational study was conducted among adults attending otolaryngology and audiology clinics in Kazakhstan between January 2023 and December 2024. Participants were recruited during routine audiological consultation or following referral from otorhinolaryngology services. The study protocol integrated standardized clinical assessment, hearing-aid fitting, immediate post-fitting evaluation, and 3-month follow-up. The analytical sample comprised 250 patients aged 18–70 years with clinically confirmed hearing impairment who underwent standardized audiological assessment and hearing aid evaluation. Eligible participants were adults aged 18–70 years with bilateral clinically confirmed conductive or sensorineural hearing loss who were candidates for bilateral hearing-aid fitting and who were able to complete speech audiometry testing in the Kazakh language. Conductive hearing loss was defined by an air–bone gap of ≥15 dB with preserved bone-conduction thresholds, whereas sensorineural hearing loss was defined by elevated air- and bone-conduction thresholds with an air–bone gap <10 dB. Only patients with stage II or stage III hearing loss were included. To reduce heterogeneity related to prolonged auditory deprivation, patients were eligible if the clinically established duration of hearing loss was between 1 and 3 years. Duration of hearing loss was assessed at baseline based on clinical history and time since established diagnosis. To reduce heterogeneity related to audiometric configuration, eligible patients had audiometric profiles suitable for conventional hearing-aid fitting.

Patients were excluded if they had mixed hearing loss, unilateral hearing loss, previous otologic surgery, neurological disease, cognitive impairment that could affect speech perception testing, insufficient Kazakh-language proficiency, prior hearing-aid use, long-standing hearing loss exceeding 3 years, sharply descending or sharply ascending audiograms, isolated high-frequency hearing loss, or inability to complete the 3-month follow-up assessment. A baseline assessment was performed at the initial consultation before hearing-aid fitting. An immediate post-fitting speech perception assessment was conducted after hearing-aid fitting and programming. The follow-up assessment was performed 3 months after hearing-aid fitting. Patients who obtained hearing aids more than one month after the initial consultation underwent repeat audiological and speech perception assessment before fitting, and this reassessment was treated as the baseline evaluation for analysis. Written informed consent was obtained from all participants prior to inclusion.

### 2.2. Audiological Assessment and Hearing Aid Fitting

All audiological assessments and hearing-aid fittings were performed by qualified audiologists according to standardized clinical protocols for the diagnosis and rehabilitation of hearing loss. Pure-tone air- and bone-conduction thresholds were measured separately for the right and left ears in a sound-treated booth using calibrated audiometric equipment. Masking was applied when clinically indicated according to standard audiological procedures. For each ear, the pure-tone average (PTA) was calculated using air-conduction thresholds at 500, 1000, 2000, and 4000 Hz. In the main analysis, tonal threshold referred to the bilateral PTA, calculated as the mean of the right- and left-ear PTAs, and is expressed in dB HL. In addition to the PTA, frequency-specific thresholds were recorded at standard audiometric frequencies and used to characterize audiometric configuration. Audiograms were categorized descriptively as flat or gradually descending based on the pattern of air-conduction thresholds across frequencies. Because all participants had bilateral hearing loss and received bilateral hearing-aid fitting, no single aided ear was defined for the main analysis.

All participants were fitted bilaterally with digital Phonak Audéo L50 receiver-in-canal hearing aids of the same technology level (Phonak, Sonova AG, Stäfa, Switzerland). Initial programming was performed using the manufacturer’s fitting software, with NAL-NL2 used as the primary prescriptive fitting rationale. This prescription was selected to optimize speech audibility while maintaining comfortable loudness for adult hearing-aid users. After the initial fitting, individualized fine-tuning was performed according to each patient’s audiometric configuration, perceived audibility, loudness comfort, and speech perception performance.

Receiver strength was selected individually according to the degree and configuration of hearing loss, following manufacturer recommendations. However, the hearing-aid model, technology level, fitting rationale, and core signal-processing features were consistent across participants. Therefore, patients with conductive and sensorineural hearing loss received comparable hearing-aid technology, including the same general signal-processing platform, directional microphone strategy, noise-reduction system, multichannel compression, and feedback-management system. Differences between participants were limited primarily to individualized gain, receiver selection, and frequency-specific amplification settings required by each patient’s audiometric profile.

Fitting adequacy was evaluated primarily through patient-reported audibility, loudness comfort, and aided speech perception testing in a free-field setting. Real-ear measurement was not performed routinely for all participants. It was used selectively when there was clinical uncertainty regarding fitting adequacy or when patients reported insufficient benefit, discomfort, or poor speech perception despite initial fine-tuning. Therefore, objective verification of aided gain against prescriptive targets was not available for the full cohort, and fitting adequacy was assessed primarily through clinical fine-tuning, patient-reported audibility and loudness comfort, and aided speech perception testing.

All patients received standardized counseling on hearing-aid use, adaptation, and expected benefits. Hearing-aid adherence during follow-up was assessed at the 3-month visit using the datalogging function available in the fitting software. Average daily wearing time was extracted in hours per day, and any adaptation problems, discomfort, or discontinuation of use were documented during follow-up. This standardized fitting and follow-up approach minimized heterogeneity related to device model, technology level, and fitting laterality, while datalogging allowed hearing-aid use during follow-up to be documented and compared between groups.

### 2.3. Speech Perception Assessment

Speech perception was assessed using a Kazakh-language speech audiometry test developed for clinical use in the study population. The test consisted of equivalent lists of phonetically balanced monosyllabic and disyllabic words. The task was administered as an open-set speech recognition test: participants did not know the presented words in advance and were asked to repeat each word aloud. Each list included 20 words, with each correctly repeated word contributing 5 percentage points to the final score. Results were expressed as the percentage of correctly recognized words.

Testing was conducted in a free-field setting under standardized acoustic conditions. For speech perception in quiet, the speech signal was presented at 60 dB SPL without background noise. For speech perception in noise, the speech signal was presented at 60 dB SPL with broadband speech-shaped noise at a fixed signal-to-noise ratio of +3 dB. Thus, 60 dB SPL referred to the speech presentation level, not the noise level; the noise level was determined by the fixed SNR. Speech perception was assessed without hearing aids at baseline, immediately after hearing-aid fitting, and at the 3-month follow-up, where applicable. All assessments were performed by trained audiology personnel using the same testing protocol.

### 2.4. Outcomes and Independent Variables

The study outcomes were speech perception scores expressed as the percentage of correctly recognized words. Six predefined outcomes were analyzed: unaided speech perception in quiet at 60 dB SPL, unaided speech perception in noise at 60 dB SPL with SNR +3 dB, aided speech perception in quiet at 60 dB SPL immediately after fitting, aided speech perception in noise at 60 dB SPL with SNR +3 dB immediately after fitting, aided speech perception in quiet at 60 dB SPL at 3 months, and aided speech perception in noise at 60 dB SPL with SNR +3 dB at 3 months.

The primary exposure variable was hearing loss type (conductive vs. sensorineural). Because the same hearing-aid model and technology level were used across the cohort, the hearing-aid device class was not included as a separate covariate. Individual fitting parameters, including gain and receiver strength, were adjusted according to each patient’s audiometric profile. Prespecified covariates included age, sex, bilateral pure-tone average threshold, hearing loss stage (II vs. III), educational attainment, presence of chronic conditions, living status (living alone vs. not), residential status (urban vs. rural), and geographic residency (Almaty vs. other regions). Duration of hearing loss was recorded at baseline and was restricted by eligibility criteria to 1–3 years to reduce heterogeneity related to prolonged auditory deprivation. In the present study, tonal threshold referred to the pure-tone average calculated across 500, 1000, 2000, and 4000 Hz in dB HL. Because all participants had bilateral hearing loss and were fitted bilaterally, the mean bilateral pure-tone average was used for the main analysis. These variables were selected a priori based on clinical relevance and potential confounding effects.

### 2.5. Statistical Analysis

All statistical analyses were performed using R version 4.5.1 (R Foundation for Statistical Computing, Vienna, Austria) [19]. Descriptive statistics were used to summarize baseline characteristics and hearing-related outcomes by hearing loss group. Continuous variables were reported as mean ± standard deviation, while categorical variables were presented as counts and percentages. Between-group differences were assessed using Welch’s two-sample t-test for continuous variables and Pearson’s chi-squared test or Fisher’s exact test for categorical variables, as appropriate. Two distinct modeling approaches were prespecified because they addressed different inferential questions: adjusted 3-month regression models estimated follow-up aided speech perception conditional on immediate post-fitting performance and baseline covariates, whereas mixed-effects models assessed within-participant change over time from immediate post-fitting to 3 months. To estimate adjusted differences in follow-up outcomes, multivariable linear regression models were fitted separately for aided speech perception in quiet at 60 dB SPL at 3 months and aided speech perception in noise at 60 dB SPL with SNR +3 dB at 3 months. Because the sensorineural hearing loss group had greater baseline hearing severity and lower immediate aided performance, the adjusted 3-month regression models included the corresponding immediate post-fitting aided speech perception score, bilateral PTA, hearing loss stage, and other prespecified demographic and clinical covariates. The immediate post-fitting aided score was included to account for pre-existing differences in early aided performance after device fitting and to estimate 3-month aided speech perception conditional on initial aided benefit. This approach was used to distinguish unadjusted between-group differences from differences that persisted after accounting for baseline auditory severity. Adjusted beta coefficients with 95% confidence intervals were reported. The purpose of these models was to estimate the association between hearing-loss type and 3-month aided speech perception after accounting for the corresponding immediate post-fitting aided speech perception score and baseline covariates. Therefore, these models should be interpreted as adjusted follow-up outcome models rather than as models of change over time. Longitudinal changes in aided speech perception from immediate post-fitting assessment to 3 months were analyzed using linear mixed-effects models with random intercepts for participants to account for within-subject correlation. Fixed effects included hearing-loss group, time, the group-by-time interaction, and prespecified demographic, clinical, and audiometric covariates. The group-by-time interaction tested whether the trajectory of aided speech perception from immediate post-fitting to 3 months differed between conductive and sensorineural hearing loss groups. Given observed baseline differences between groups, propensity score matching was additionally performed as a sensitivity analysis to assess the robustness of findings. This matched analysis was not considered the primary inferential framework. Propensity scores were estimated using logistic regression, including age, sex, bilateral PTA, hearing loss stage, education, chronic conditions, living status, residential status, and geographic residency. Nearest-neighbor matching was then performed using a 1:1 matching ratio without replacement and a caliper of 0.2 standard deviations of the logit of the propensity score. Covariate balance before and after matching was evaluated using standardized mean differences. Statistical significance was defined as a two-sided *p*-value <0.05.

### 2.6. Ethical Considerations

The study was conducted in accordance with the principles of the Declaration of Helsinki. Ethical approval was obtained from the Local Ethics Committee of Asfendiyarov Kazakh National Medical University (4 September 2023, protocol No. 154). All participants provided written informed consent prior to participation. Patient data were anonymized prior to analysis to ensure confidentiality and data protection. All data analyzed in the present study are provided in Supplemental Table S1.

## 3. Results

Table 1 presents descriptive characteristics of study participants. A total of 250 patients were included in the analysis, of whom 100 (40%) had conductive hearing loss, and 150 (60%) had sensorineural hearing loss. The overall mean age of the study population was 47.76 ± 11.44 years, with no statistically significant difference between the two groups (48.46 ± 10.41 vs. 47.29 ± 12.09 years, *p* = 0.4). Similarly, the sex distribution was comparable, with males comprising 58% of the overall sample and no significant between-group difference (*p* = 0.4). In contrast, several clinically relevant differences were observed between the groups. Patients with sensorineural hearing loss had significantly higher mean tonal thresholds compared with those with conductive hearing loss (54.24 ± 7.07 vs. 51.05 ± 5.71 dB HL, *p* < 0.001), indicating greater severity of auditory impairment. This difference was further reflected in the distribution of hearing loss stages: stage III hearing loss was more prevalent among patients with sensorineural hearing loss (39%) compared with those with conductive hearing loss (26%), whereas stage II hearing loss predominated in the conductive group (74% vs. 61%; *p* = 0.029). Educational attainment also differed markedly between groups. A substantially higher proportion of patients with sensorineural hearing loss had a bachelor’s degree or higher (75% vs. 45%), while individuals with conductive hearing loss were more likely to have only a high school education (48% vs. 12%) (*p* < 0.001). Geographic distribution showed similar disparities, with patients residing in Almaty more frequently represented in the sensorineural group (35% vs. 15%), whereas the majority of patients with conductive hearing loss were from other regions (85% vs. 65%) (*p* < 0.001). In contrast, no significant differences were observed in the prevalence of chronic conditions, living status, urban versus rural residence, or average daily hearing-aid wearing time, suggesting broadly comparable distributions of these factors across groups. These baseline differences indicate that patients with sensorineural hearing loss had greater auditory impairment at study entry. Therefore, subsequent analyses were interpreted with caution and adjusted for tonal threshold and hearing loss stage to reduce confounding by baseline hearing severity.

**Figure 1:**
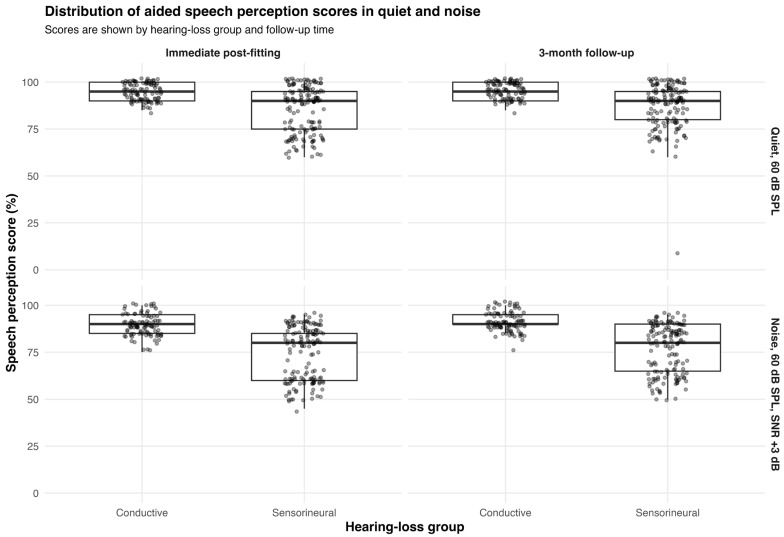
Distribution of aided speech perception scores in quiet and noise by hearing-loss group and time.

Speech perception scores are presented in Table 2, and aided speech perception scores are illustrated in Figure 1. Unadjusted analyses demonstrated consistently higher speech perception scores among patients with conductive hearing loss compared with those with sensorineural hearing loss across all assessed outcomes. At baseline, patients with conductive hearing loss had significantly higher unaided speech perception in quiet at 60 dB SPL than patients with sensorineural hearing loss (72.55 ± 8.21 vs. 64.23 ± 13.41, *p* < 0.001). Similarly, unaided speech perception in noise at 60 dB SPL with SNR +3 dB was significantly higher in the conductive hearing loss group than in the sensorineural hearing loss group (57.55 ± 8.21 vs. 48.80 ± 14.71, *p* < 0.001). Following the hearing-aid fitting, the between-group difference remained evident. Aided speech perception in quiet at 60 dB SPL was significantly higher among patients with conductive hearing loss than among those with sensorineural hearing loss (94.80 ± 4.66 vs. 85.20 ± 12.33, *p* < 0.001). A similar pattern was observed for aided speech perception in noise at 60 dB SPL with SNR +3 dB, with higher scores in the conductive group compared with the sensorineural group (89.50 ± 5.71 vs. 74.30 ± 14.26, *p* < 0.001). At the 3-month follow-up, both groups demonstrated high aided speech perception scores; however, the between-group difference persisted. Patients with conductive hearing loss maintained significantly higher aided speech perception in quiet at 60 dB SPL at 3 months compared with patients with sensorineural hearing loss (95.80 ± 4.25 vs. 87.11 ± 12.05, *p* < 0.001). They also had significantly higher aided speech perception in noise at 60 dB SPL with SNR +3 dB at 3 months (91.35 ± 5.12 vs. 76.80 ± 12.90, *p* < 0.001). Overall, these findings indicate that although aided speech perception was high in both groups after hearing-aid fitting, patients with sensorineural hearing loss consistently showed lower speech perception performance across all unaided and aided outcomes, particularly under noise conditions.

Two complementary modeling approaches were used, but they addressed different inferential questions. First, the adjusted 3-month linear regression models estimated the between-group differences in follow-up aided speech perception after conditioning on the corresponding immediate post-fitting aided score and prespecified covariates. These models, therefore, evaluated adjusted 3-month outcomes rather than change over time. Second, longitudinal mixed-effects models evaluated the repeated aided speech perception scores from immediate post-fitting to 3 months, estimating overall group differences, time effects, and group-by-time interactions. The mixed-effects models, therefore, assessed whether aided speech perception changed during follow-up and whether this change differed between the conductive and sensorineural hearing loss groups. In the adjusted 3-month linear regression models, the corresponding immediate post-fitting aided speech perception score was included to account for differences in early aided benefit after fitting and was the strongest predictor of 3-month outcomes in both aided testing conditions (Table 3). For aided speech perception in quiet at 60 dB SPL at 3 months, higher immediate post-fitting aided speech perception at 60 dB was strongly associated with higher follow-up scores (β = 0.94; 95% CI: 0.80 to 1.07; *p* < 0.001). Similarly, for aided speech perception in noise at 60 dB SPL with SNR +3 dB at 3 months, higher immediate post-fitting aided speech perception in noise was strongly associated with follow-up performance (β = 0.86; 95% CI: 0.79 to 0.93; *p* < 0.001). After adjustment for baseline severity and other covariates, hearing loss type was not significantly associated with 3-month aided speech perception in quiet at 60 dB SPL conditions; patients with sensorineural hearing loss had slightly lower adjusted scores than those with conductive hearing loss, but the difference was not statistically significant (β = −0.71; 95% CI: −2.47 to 1.05; *p* = 0.427). However, sensorineural hearing loss remained independently associated with poorer 3-month aided speech perception in noise compared with conductive hearing loss (β = −1.73; 95% CI: −3.10 to −0.36; *p* = 0.014). Tonal threshold and hearing loss stage were not independently associated with 3-month outcomes after adjustment for either aided speech perception in quiet at 60 dB SPL or aided speech perception in noise at 60 dB SPL with SNR +3 dB. These adjusted 3-month regression findings suggest that part of the unadjusted between-group difference in follow-up outcomes was explained by immediate post-fitting aided performance, baseline hearing severity, and other covariates, whereas the adjusted 3-month difference in aided speech perception in noise remained statistically significant.

**Figure 2:**
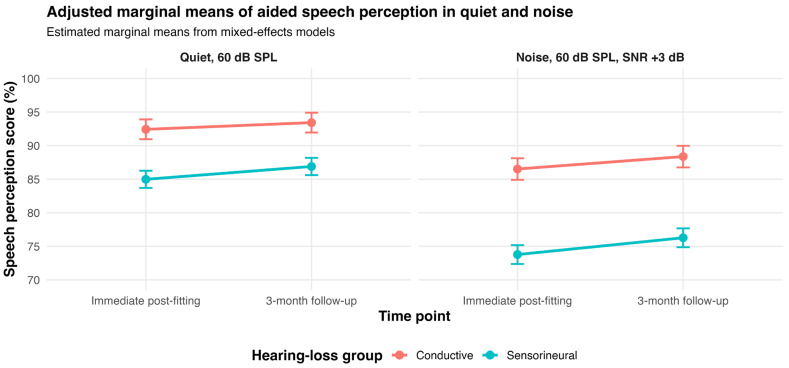
Adjusted marginal means of aided speech perception in quiet and noise from immediate post-fitting to 3 months.

Figure 2 presents the adjusted marginal means of aided speech perception outcomes over time, derived from mixed-effects models controlling for demographic, audiometric, and clinical covariates. Across both aided testing conditions, patients with conductive hearing loss consistently demonstrated higher adjusted speech perception scores than patients with sensorineural hearing loss at both the immediate post-fitting assessment and the 3-month follow-up. For aided speech perception in 60 dB SPL noise with an SNR of +3 dB, adjusted scores increased modestly over time in both groups, indicating improvement after continued hearing-aid use. However, a persistent between-group gap remained, with the conductive hearing loss group showing higher adjusted scores at both time points. A similar pattern was observed for aided speech perception in quiet at 60 dB SPL, although overall scores were higher than in the noise condition, and patients with conductive hearing loss demonstrated near-ceiling performance. These findings suggest that hearing aids were associated with improved aided speech perception over time but did not fully eliminate the performance gap between conductive and sensorineural hearing loss groups.

Unlike the adjusted 3-month regression models, the mixed-effects models assessed repeated aided speech perception scores from immediate post-fitting to 3 months and tested whether the trajectory of change differed by hearing-loss type. In longitudinal mixed-effects models, several factors were significantly associated with changes in aided speech perception over time, with consistent patterns observed across both quiet and noise conditions (Table 4). For speech perception in quiet, patients with sensorineural hearing loss demonstrated significantly lower performance compared with those with conductive hearing loss (β = −7.45; 95% CI: −8.90 to −5.99; *p* < 0.001). Increasing age was independently associated with lower speech perception scores (β = −0.16 per year; 95% CI: −0.21 to −0.10; *p* < 0.001), as was higher tonal threshold (β = −0.59; 95% CI: −0.76 to −0.42; *p* < 0.001), indicating that greater severity of hearing impairment was linked to poorer outcomes. Similarly, patients with more advanced hearing loss (stage III) had significantly lower speech perception compared with those with stage II (β = −6.80; 95% CI: −9.07 to −4.53; *p* < 0.001). Social factors also emerged as relevant predictors: individuals living alone (β = −1.59; 95% CI: −2.98 to −0.21; *p* = 0.024) and those residing in urban areas (β = −1.66; 95% CI: −3.07 to −0.25; *p* = 0.021) had lower longitudinal speech perception scores. No statistically significant overall improvement over time was observed in quiet conditions (*p* = 0.156), and the interaction between hearing loss type and time was not significant, indicating similar trajectories of change across groups. In noise conditions, similar but more pronounced patterns were observed. Sensorineural hearing loss was associated with substantially lower speech perception scores compared with conductive hearing loss (β = −12.74; 95% CI: −14.26 to −11.23; *p* < 0.001). Unlike quiet conditions, a modest but statistically significant improvement over time was detected (β = 1.85; 95% CI: 0.44 to 3.26; *p* = 0.010), suggesting measurable adaptation to hearing aid use in more challenging listening environments. Age (β = −0.18; 95% CI: −0.24 to −0.12; *p* < 0.001), higher tonal threshold (β = −0.70; 95% CI: −0.88 to −0.52; *p* < 0.001), and stage III hearing loss (β = −6.76; 95% CI: −9.19 to −4.34; *p* < 0.001) were again independently associated with poorer outcomes. Living alone was also a significant predictor of reduced speech perception in noise (β = −2.30; 95% CI: −3.81 to −0.80; *p* = 0.003). As in quiet conditions, the interaction between hearing loss type and time was not statistically significant, indicating that the amount of change from immediate post-fitting to 3 months did not differ significantly between conductive and sensorineural hearing loss groups.

**Figure 3:**
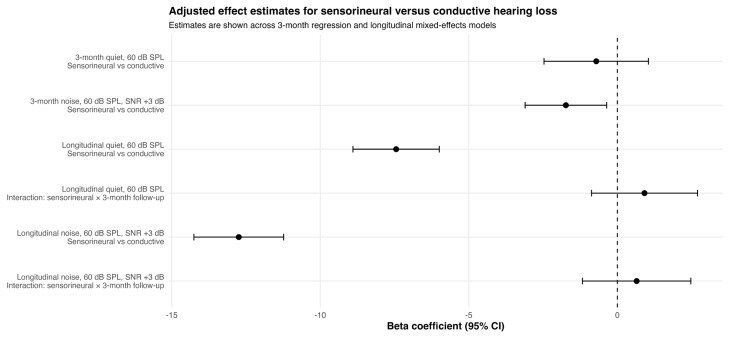
Adjusted effect estimates for sensorineural versus conductive hearing loss across 3-month regression and longitudinal mixed-effects models.

Figure 3 presents the adjusted effect estimates for the association between hearing loss type and aided speech perception outcomes across the primary 3-month adjusted models and longitudinal mixed-effects models. In the longitudinal models, sensorineural hearing loss was consistently associated with lower aided speech perception performance compared with conductive hearing loss. This association was particularly pronounced for aided speech perception in noise at 60 dB SPL with SNR +3 dB, where sensorineural hearing loss was associated with a substantial reduction in speech perception scores, and the confidence interval did not cross the null. A similarly strong, although slightly smaller, negative association was observed for aided speech perception in quiet at 60 dB SPL, indicating that the performance disadvantage associated with sensorineural hearing loss was present across both aided testing conditions.

In contrast, the adjusted 3-month models showed a more nuanced pattern. For aided speech perception in noise at 60 dB SPL with SNR +3 dB at 3 months, sensorineural hearing loss remained significantly associated with lower speech perception performance compared with conductive hearing loss, although the magnitude of the adjusted effect was smaller than in the longitudinal model. For aided speech perception in quiet at 60 dB SPL at 3 months, the adjusted difference between groups was not statistically significant, with the confidence interval crossing the null. This suggests that baseline aided speech perception, tonal threshold, hearing loss stage, and other covariates largely accounted for the between-group difference in aided speech perception in quiet at 60 dB SPL at 3 months. Importantly, the interaction terms between hearing loss type and time were not statistically significant for either aided speech perception in quiet at 60 dB SPL or aided speech perception in noise at 60 dB SPL with SNR +3 dB. This indicates that although aided speech perception changed over time, the relative difference between conductive and sensorineural hearing loss groups remained broadly stable during the 3-month follow-up period.

**Figure 4:**
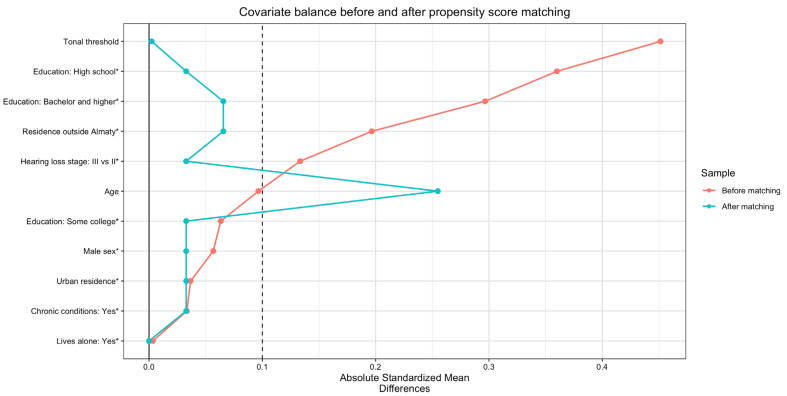
Covariate balance before and after propensity score matching in the secondary sensitivity analysis.

Propensity score matching was performed as a sensitivity analysis to assess the robustness of the findings after reducing baseline imbalance between the conductive and sensorineural hearing loss groups. Using 1:1 nearest-neighbor matching without replacement and a caliper of 0.2, 122 patients were retained in the matched sample, including 61 patients with conductive hearing loss and 61 patients with sensorineural hearing loss. Covariate balance improved after matching, with standardized mean differences reduced for the main imbalanced covariates, including pure-tone average, education, residency, and hearing loss stage (Figure 4). In the matched analysis, sensorineural hearing loss was not significantly associated with aided speech perception in quiet at 60 dB SPL at 3 months (β = −1.33; 95% CI: −3.90 to 1.25; *p* = 0.310), but remained significantly associated with poorer aided speech perception in noise at 60 dB SPL with SNR +3 dB at 3 months (β = −1.76; 95% CI: −2.97 to −0.54; *p* = 0.005). These sensitivity findings were consistent with the primary adjusted 3-month regression results, supporting the robustness of the conclusion that the between-group difference was most evident under noise conditions. However, the matched analysis was interpreted as confirmatory sensitivity evidence rather than as the primary basis for inference.

## 4. Discussion

This study provides a comprehensive evaluation of hearing aid–related speech perception outcomes among adults with conductive and sensorineural hearing loss in Kazakhstan, addressing an important gap in evidence from Central Asian clinical settings. The findings demonstrate three key patterns. First, unadjusted analyses showed higher speech perception scores among patients with conductive hearing loss than among those with sensorineural hearing loss across all assessed conditions. Second, the adjusted 3-month regression models showed that the between-group difference was no longer statistically significant for aided speech perception in quiet at 60 dB SPL after conditioning on immediate post-fitting performance and covariates, whereas a smaller but statistically significant difference remained for aided speech perception in noise. Third, the mixed-effects models showed lower overall aided speech perception among patients with sensorineural hearing loss and demonstrated modest improvement over time only for speech perception in noise; however, the group-by-time interaction was not statistically significant. In the mixed-effects model for aided speech perception in quiet at 60 dB SPL, poorer performance was associated with sensorineural hearing loss, older age, higher tonal threshold, more advanced hearing loss stage, living alone, and urban residence. However, the association with urban residence was not consistent across all models or testing conditions and should therefore be interpreted cautiously. In noise conditions, poorer performance was independently associated with sensorineural hearing loss, older age, higher tonal threshold, more advanced hearing loss stage, and living alone, whereas speech perception improved modestly over time in both groups. Taken together, these findings suggest that aided speech perception outcomes were associated not only with hearing loss type but also with auditory severity, age, and selected social-contextual characteristics, although the observational design limits causal interpretation.

An important consideration is that the sensorineural hearing loss group had greater baseline hearing severity, as reflected by higher pure-tone thresholds and a higher proportion of stage III hearing loss. Therefore, the poorer unadjusted speech perception outcomes observed in this group should not be interpreted as being attributable to hearing-loss type alone. In the adjusted 3-month regression models, conditioning on immediate post-fitting aided performance, tonal threshold, hearing loss stage, and other covariates attenuated the between-group difference in the 60 dB SPL condition, while the adjusted 3-month difference in aided speech perception in noise remained statistically significant. This pattern suggests that baseline severity partly explained the observed differences, whereas speech-in-noise perception remained more vulnerable among patients with sensorineural hearing loss. The use of the same hearing-aid model, technology level, and fitting rationale across groups reduces heterogeneity related to device class and signal-processing platform. However, because real-ear measurement was not routinely performed, the study could not confirm objective aided gain against prescriptive targets for all participants. Therefore, residual variability in fitting accuracy may have contributed to individual differences in aided speech perception and should be considered when interpreting between-group differences. Nevertheless, individual fitting parameters necessarily differed according to audiometric configuration, and residual variation in amplification settings may still have contributed to individual differences in aided speech perception. By contrast, the mixed-effects models addressed a separate longitudinal question: whether aided speech perception changed from immediate post-fitting to 3 months and whether this change differed by hearing-loss type. These models showed a significant overall time effect only for speech perception in noise and no statistically significant group-by-time interaction, suggesting that early change during follow-up was broadly similar across hearing-loss groups.

The observed differences in speech perception outcomes between conductive and sensorineural hearing loss are consistent with fundamental auditory pathophysiology and provide clinically meaningful insights into rehabilitation strategies [20,21]. In conductive hearing loss, cochlear and neural processing mechanisms are largely preserved, and amplification primarily compensates for reduced sound transmission through the outer and middle ear. As a result, once audibility is restored, patients are typically able to achieve near-normal speech perception, particularly in quiet conditions [22,23]. This is reflected in the present study by the near-ceiling performance observed in the conductive group following hearing aid fitting. In contrast, sensorineural hearing loss is characterized by damage to cochlear hair cells and, in many cases, disruption of neural encoding processes. These deficits lead not only to reduced audibility but also to impaired frequency selectivity, temporal resolution, and neural synchrony. Consequently, even when audibility is partially restored through amplification, the quality of the auditory signal remains degraded. This limitation is particularly evident in complex listening environments, where speech perception depends on the ability to segregate signal from background noise and to process fine spectral and temporal cues. The substantially lower performance observed among patients with sensorineural hearing loss in noise conditions, even after adjustment for confounders, is therefore consistent with well-established auditory processing constraints [24,25].

Importantly, the finding that immediate post-fitting aided speech perception strongly predicted 3-month outcomes highlights the role of early aided benefit as a key determinant of short-term rehabilitation outcomes [26,27]. This association should not be interpreted as evidence that early aided performance causes later outcomes; rather, it indicates that patients who achieved better aided speech perception immediately after fitting also tended to have better aided speech perception at follow-up. From an audiological perspective, this suggests that initial aided performance may serve as a practical clinical indicator for identifying patients at risk of suboptimal long-term outcomes. Such patients may benefit from more intensive rehabilitation strategies, including auditory training, fine-tuning of device parameters, or the use of advanced signal-processing features such as directional microphones and noise reduction algorithms.

Beyond audiological factors, the findings suggest that selected demographic and social characteristics may be associated with aided speech perception outcomes. Increasing age, higher tonal thresholds, and a more advanced hearing loss stage were consistently associated with poorer speech perception, reflecting the cumulative influence of biological and sensory factors. Living alone was also associated with lower aided speech perception in the mixed-effects models, particularly under noise conditions. This association may reflect fewer daily communication opportunities, reduced social reinforcement for consistent hearing-aid use, less family support during adaptation, or lower engagement with follow-up rehabilitation. However, these mechanisms were not directly measured and should be interpreted as plausible explanations rather than confirmed causal pathways. Similarly, the observed association between urban residence and poorer aided speech perception in quiet at 60 dB SPL should be interpreted cautiously because it was not consistent across all outcomes and may reflect residual confounding by unmeasured contextual, socioeconomic, clinical, or service-related factors.

This social dimension is particularly relevant in the context of Kazakhstan, where traditional cultural norms have historically emphasized multigenerational living, with older adults residing with their children and benefiting from continuous social and communicative support [28,29]. However, recent societal changes, including urbanization, labor migration, and shifting family structures, have led to a growing proportion of elderly individuals living independently. As these norms evolve, older adults increasingly rely on their own functional abilities, including hearing, to maintain communication, safety, and social participation. The present findings are consistent with the possibility that changing living arrangements may be relevant to hearing rehabilitation outcomes, particularly among individuals living alone. However, this study did not directly measure family support, communication frequency, or daily listening environments; therefore, these contextual explanations should be considered hypothesis-generating.

These demographic and cultural shifts coincide with ongoing healthcare reforms in Kazakhstan aimed at improving access to audiological care. The introduction of mandatory social health insurance and broader efforts to modernize service delivery have been accompanied by a policy shift toward expanding access to hearing technologies across the population [30,31]. This process has been further strengthened by recent legislative changes (Law of the Republic of Kazakhstan No. 185-VIII, 25 April 2025), which enable local executive bodies to fund hearing aids and speech processors for patients without formal disability status based on medical indications [32]. While these reforms represent a significant step toward equitable access, the present findings suggest that access to hearing devices alone may not fully address variability in aided speech perception outcomes.

Taken together, the findings of this study underscore the need for healthcare systems to adapt not only by expanding the provision of hearing devices but also by addressing the broader clinical and social determinants of successful rehabilitation. These findings support the potential value of implementing standardized outcome assessments (e.g., speech perception testing in both quiet and noise conditions), alongside structured follow-up protocols and patient-centered counseling. In addition, targeted support strategies should be developed for vulnerable populations, particularly older adults living alone. In parallel, future research and service planning should consider whether living arrangements, communication support, rehabilitation engagement, and local service context influence hearing-aid outcomes, because these factors were not directly measured in the present study.

Several limitations should be considered when interpreting these findings. First, although this was a prospective longitudinal observational study with follow-up assessment, it did not involve random allocation; therefore, causal inference is limited, and residual confounding cannot be excluded. Second, real-ear measurement was not performed routinely for all participants, and objective verification of aided gain against prescriptive targets was not available for the full cohort. This is an important limitation because fitting accuracy can directly influence aided audibility, loudness comfort, and speech perception outcomes, particularly in noise. Although all patients received the same hearing-aid model, technology level, and NAL-NL2-based fitting rationale, individualized fine-tuning without routine real-ear verification may have resulted in residual variation in achieved aided gain across participants. Therefore, some of the observed variability in aided speech perception, including between-group differences, may partly reflect differences in fitting accuracy rather than hearing-loss type or patient-level characteristics alone. Third, the study was conducted in clinical audiology settings in Kazakhstan, which may limit generalizability to other healthcare contexts. Fourth, the 3-month follow-up period may not fully capture long-term adaptation to hearing aids, particularly in patients with sensorineural hearing loss. Fifth, although living alone and, in one model, urban residence were associated with poorer aided speech perception, communication frequency, family involvement, rehabilitation engagement, social support, socioeconomic status, daily listening environments, and differences in local service context were not directly measured. Therefore, residual confounding by unmeasured contextual factors cannot be excluded.

## 5. Conclusions

These findings suggest that differences in aided speech perception between conductive and sensorineural hearing loss are partly influenced by baseline hearing severity, while speech-in-noise performance may remain poorer among patients with sensorineural hearing loss even after adjustment. However, the findings of our study should be interpreted cautiously and should not be considered causal. Routine assessment of speech perception in noise, together with careful consideration of hearing severity and social-contextual factors, may help optimize hearing rehabilitation. Future policy and clinical practice should move toward comprehensive care models that combine audiological, social, and rehabilitative support. Such an approach will be essential to maximize the functional benefits of hearing aids, reduce disparities in outcomes, and ensure that expanding access translates into meaningful improvements in quality of life.

## Figures and Tables

**Table 1:** Descriptive characteristics by hearing loss group.

Variable	Conductive N = 100	Sensorineural N = 150	*p*-Value	Overall N = 250
Age, Mean ± SD	48.46 ± 10.41	47.29 ± 12.09	0.4	47.76 ± 11.44
Gender, *n* (%)			0.4	
Female	39 (39%)	67 (45%)		106 (42%)
Male	61 (61%)	83 (55%)		144 (58%)
Pure-tone average, dB HL, Mean ± SD	51.05 ± 5.71	54.24 ± 7.07	<0.001	52.96 ± 6.73
Hearing loss stage, *n* (%)			0.029	
II	74 (74%)	91 (61%)		165 (66%)
III	26 (26%)	59 (39%)		85 (34%)
Education, *n* (%)			<0.001	
Bachelor’s and higher	45 (45%)	112 (75%)		157 (63%)
High school	48 (48%)	18 (12%)		66 (26%)
Some college	7 (7.0%)	20 (13%)		27 (11%)
Chronic conditions (yes), *n* (%)	34 (34%)	46 (31%)	0.6	80 (32%)
Lives alone (yes), *n* (%)	21 (21%)	32 (21%)	>0.9	53 (21%)
Resident status, *n* (%)			0.6	
Rural	51 (51%)	71 (47%)		122 (49%)
Urban	49 (49%)	79 (53%)		128 (51%)
Residency, *n* (%)			<0.001	
Almaty	15 (15%)	52 (35%)		67 (27%)
Other	85 (85%)	98 (65%)		183 (73%)
Average daily hearing-aid wearing time, hours/day, Mean ± SD	6.39 ± 1.08	6.19 ± 1.14	0.17	6.27 ± 1.12

**Table 2:** Unadjusted speech perception outcomes by hearing loss group.

Outcome	Conductive N = 100, Mean ± SD	Sensorineural N = 150, Mean ± SD	*p*-Value	Overall N = 250, Mean ± SD
Unaided speech perception in quiet at 60 dB SPL	72.55 ± 8.21	64.23 ± 13.41	<0.001	67.56 ± 12.29
Unaided speech perception in noise at 60 dB SPL with SNR +3 dB	57.55 ± 8.21	48.80 ± 14.71	<0.001	52.30 ± 13.22
Aided speech perception in quiet at 60 dB SPL, immediate post-fitting	94.80 ± 4.66	85.20 ± 12.33	<0.001	89.04 ± 11.03
Aided speech perception in noise at 60 dB SPL with SNR +3 dB, immediate post-fitting	89.50 ± 5.71	74.30 ± 14.26	<0.001	80.38 ± 13.80
Aided speech perception in quiet at 60 dB SPL at 3 months	95.80 ± 4.25	87.11 ± 12.05	<0.001	90.59 ± 10.60
Aided speech perception in noise at 60 dB SPL with SNR +3 dB at 3 months	91.35 ± 5.12	76.80 ± 12.90	<0.001	82.62 ± 12.69

**Table 3:** Adjusted linear regression models for 3-month aided speech perception outcomes.

Term	Adjusted Beta (95% CI)	SE	t	*p* Value
3-month aided speech perception in quiet at 60 dB SPL				
Aided speech perception in quiet at 60 dB SPL, immediate post-fitting	0.94 (0.80 to 1.07)	0.067	13.933	<0.001
Group (Ref. Conductive) Sensorineural	−0.71 (−2.47 to 1.05)	0.892	−0.796	0.427
Age	−0.01 (−0.08 to 0.05)	0.034	−0.452	0.652
Gender (Ref. Female) Male	0.79 (−0.53 to 2.10)	0.667	1.178	0.240
Tonal threshold	0.18 (−0.05 to 0.40)	0.113	1.564	0.119
Hearing loss (Ref. Level II) Level III	0.74 (−2.25 to 3.72)	1.514	0.487	0.627
Education (Ref. Bachelor and higher) High school	−0.36 (−2.03 to 1.30)	0.845	−0.432	0.666
Some college	0.27 (−1.94 to 2.48)	1.120	0.242	0.809
Chronic conditions (Ref. No) Yes	0.40 (−1.04 to 1.83)	0.731	0.541	0.589
Lives alone (Ref. No) Yes	−0.04 (−1.68 to 1.60)	0.833	−0.045	0.964
Resident status (Ref. Rural) Urban	−1.53 (−3.20 to 0.13)	0.845	−1.815	0.071
Residency (Ref. Almaty) Other	−1.50 (−3.37 to 0.37)	0.950	−1.579	0.116
3-month aided speech perception in noise at 60 dB SPL with SNR +3 dB				
Aided speech perception in noise at 60 dB SPL with SNR +3 dB, immediate post-fitting	0.86 (0.79 to 0.93)	0.035	24.362	<0.001
Group (Ref. Conductive) Sensorineural	−1.73 (−3.10 to −0.36)	0.697	−2.486	0.014
Age	0.00 (−0.04 to 0.05)	0.024	0.151	0.880
Gender (Ref. Female) Male	−0.20 (−1.12 to 0.73)	0.471	−0.418	0.677
Tonal threshold	0.03 (−0.12 to 0.18)	0.078	0.388	0.698
Hearing loss (Ref. Level II) Level III	−0.14 (−2.19 to 1.92)	1.044	−0.133	0.894
Education (Ref. Bachelor and higher) High school	−0.02 (−1.19 to 1.16)	0.597	−0.030	0.976
Some college	−0.42 (−1.98 to 1.14)	0.791	−0.534	0.594
Chronic conditions (Ref. No) Yes	0.12 (−0.90 to 1.14)	0.517	0.226	0.821
Lives alone (Ref. No) Yes	−0.51 (−1.67 to 0.65)	0.587	−0.869	0.386
Resident status (Ref. Rural) Urban	−1.11 (−2.29 to 0.06)	0.597	−1.864	0.064
Residency (Ref. Almaty) Other	−0.99 (−2.31 to 0.33)	0.671	−1.473	0.142

**Table 4:** Mixed-effects models of longitudinal change in aided speech perception from immediate post-fitting to 3 months.

Term	Beta (95% CI)	SE	t	*p* Value
Longitudinal aided speech perception in quiet at 60 dB SPL				
Group (Ref. Conductive) Sensorineural	−7.45 (−8.90 to −5.99)	0.739	−10.073	<0.001
Time: 3 months vs. immediate post-fitting	1.00 (−0.38 to 2.38)	0.703	1.423	0.156
Age	−0.16 (−0.21 to −0.10)	0.028	−5.711	<0.001
Gender (Ref. Female) Male	−0.15 (−1.28 to 0.98)	0.575	−0.260	0.795
Tonal threshold	−0.59 (−0.76 to −0.42)	0.086	−6.852	<0.001
Hearing loss (Ref. Level II) Level III	−6.80 (−9.07 to −4.53)	1.155	−5.891	<0.001
Education (Ref. Bachelor and higher) High school	−0.68 (−2.12 to 0.75)	0.732	−0.935	0.350
Some college	1.74 (−0.06 to 3.53)	0.914	1.901	0.058
Chronic conditions (Ref. No) Yes	−0.07 (−1.27 to 1.14)	0.615	−0.105	0.916
Lives alone (Ref. No) Yes	−1.59 (−2.98 to −0.21)	0.706	−2.259	0.024
Resident status (Ref. Rural) Urban	−1.66 (−3.07 to −0.25)	0.718	−2.311	0.021
Residency (Ref. Almaty) Other	−0.28 (−1.87 to 1.31)	0.808	−0.346	0.729
Group Sensorineural: Time 3 months	0.91 (−0.87 to 2.70)	0.907	1.007	0.315
Longitudinal aided speech perception in noise at 60 dB SPL with SNR +3 dB				
Group (Ref. Conductive) Sensorineural	−12.74 (−14.26 to −11.23)	0.769	−16.579	<0.001
Time: 3 months vs. immediate post-fitting	1.85 (0.44 to 3.26)	0.718	2.575	0.010
Age	−0.18 (−0.24 to −0.12)	0.030	−5.825	<0.001
Gender (Ref. Female) Male	−0.38 (−1.60 to 0.84)	0.623	−0.609	0.543
Tonal threshold	−0.70 (−0.88 to −0.52)	0.092	−7.561	<0.001
Hearing loss (Ref. Level II) Level III	−6.76 (−9.19 to −4.34)	1.233	−5.486	<0.001
Education (Ref. Bachelor and higher) High school	−0.63 (−2.19 to 0.92)	0.791	−0.802	0.423
Some college	1.91 (−0.01 to 3.82)	0.975	1.957	0.051
Chronic conditions (Ref. No) Yes	−1.13 (−2.43 to 0.17)	0.662	−1.700	0.090
Lives alone (Ref. No) Yes	−2.30 (−3.81 to −0.80)	0.765	−3.011	0.003
Resident status (Ref. Rural) Urban	−1.35 (−2.87 to 0.17)	0.772	−1.751	0.081
Residency (Ref. Almaty) Other	0.22 (−1.49 to 1.93)	0.871	0.253	0.801
Sensorineural × 3 months interaction	0.65 (−1.17 to 2.47)	0.927	0.701	0.484

## Data Availability

The original contributions presented in this study are included in the article and Supplementary Materials. Further inquiries can be directed to the corresponding author.

## Supplementary Materials

The following supporting information can be downloaded at: https://www.mdpi.com/article/10.3390/audiolres16030086/s1, Table S1: Original data analyzed for the present analysis.
